# Maßnahmen und Lösungen zur Arbeitsgestaltung für den Umgang mit der COVID-19 Pandemie: Eine systematische Analyse der Arbeit im Primär‑, Sekundär- und Tertiärsektor in Deutschland

**DOI:** 10.1007/s41449-021-00274-7

**Published:** 2021-10-13

**Authors:** Caroline Adam, Klaus Bengler, Christopher Brandl, Verena Nitsch, Gritt Ott, Sebastian Pütz, Martin Schmauder

**Affiliations:** 1 Lehrstuhl für Ergonomie, Technische Universität München, Boltzmannstraße 15, 85748 Garching b. München, Deutschland; 2 Institut für Arbeitswissenschaft, RWTH Aachen University, Eilfschornsteinstr. 18, 52056 Aachen, Deutschland; 3 Fraunhofer-Institut für Kommunikation, Informationsverarbeitung und Ergonomie FKIE, Aachen, Deutschland; 4 CIMTT Zentrum für Produktionstechnik und Organisation, Technische Universität Dresden, Helmholtzstraße 10, 01069 Dresden, Deutschland; 5 Professur für Arbeitswissenschaft, Technische Universität Dresden, Helmholtzstraße 10, 01069 Dresden, Deutschland

**Keywords:** COVID-19, Arbeit, Maßnahmen, Veränderungsprozess, Szenarien, COVID-19, Work, Measures, Change Process, Scenario

## Abstract

Die mit der Ausbreitung der COVID-19 Pandemie in Deutschland einhergehende Anforderung betriebliche Arbeitsabläufe an die gesetzlichen Hygiene- und Kontaktreduktionsvorgaben anzupassen, hat zu kurzfristigen und teilweise radikalen Veränderungen in der Arbeitswelt geführt. Im Rahmen des Forschungsvorhabens COVID19LL wurde basierend auf 52 Interviews mit Unternehmens- und Organisationsvertretungen analysiert, welche pandemiebedingten Veränderungen im Kontext der Arbeit überregional und sektorenübergreifend auftreten, wie diese zu bewerten sind und welche neuen Arbeitsweisen sich auch in einer postpandemischen Zeit bewähren könnten. Im Einklang mit anderen Untersuchungen konnten eine steigende Flexibilisierung von Arbeitsort und Arbeitszeit, eine Beschleunigung der Digitalisierung von Arbeitsprozessen sowie Auswirkungen auf die Führungskultur festgestellt werden. Weitere zentrale Erfahrungen beziehen sich auf die Anpassung interner und externer Kommunikationsprozesse sowie den operativen Umgang mit krisenbedingten Herausforderungen. Anhand der gesammelten Daten wurden Handlungsempfehlungen für Unternehmen in Form von normativ-narrativen Szenarien abgeleitet.

*Praktische Relevanz*: Die vorgestellten Ergebnisse zeigen in der Praxis erfolgreich umgesetzte Maßnahmen zur Bewältigung der krisenbedingten Herausforderungen sowie Ansätze für die künftige Weiterentwicklung von Arbeitsformen. Die Maßnahmen werden durch die Darlegung erforderlicher Rahmenbedingungen und Erfolgsfaktoren kontextualisiert, wodurch der Transfer in andere Unternehmen und Wirtschaftszweige gefördert wird.

## Abstract (en)

The demand to adapt the work processes to hygiene- and contact reduction requirements in order to curtail the spread of the COVID-19 pandemic in Germany has led to short-term and sometimes radical changes in companies and organizations. Based on 52 expert interviews with company and organization representatives, this research project analyzed which pandemic-related changes in the context of work occur on a supraregional and cross-sectoral level, how they are to be evaluated, and which new ways of working might also prove beneficial to companies after the pandemic. In line with existing studies, an increasing flexibilization of work location and working hours, an acceleration of the digitalization of work processes, and effects on leadership culture were identified. Other key experiences relate to the adaptation of internal and external communication processes and the operational handling of crisis-related challenges. Based on the results, good practices were derived in form of normative-narrative use-case based scenarios.

*Practical Relevance*: Dealing with the COVID-19 pandemic has been the central challenge for many companies over the past year and a half. The presented results show measures that have been successfully implemented in practice to overcome the challenges posed by the crisis, as well as approaches for the future development of forms of work. The measures are contextualized by outlining necessary framework conditions and success factors, thus promoting transfer to other companies and sectors of the economy.

## Einleitung

Radikale Umbrüche im Kontext der Arbeit können häufig auf technische Innovationen zurückgeführt werden. Beispielsweise ging mit der Erfindung der Dampfmaschine, der ersten industriellen Revolution, die Möglichkeit einher, Muskelkraft durch Maschinen zu ersetzen (Bubb 2006). Durch die Bereitstellung der notwendigen Infrastruktur eröffnete Elektrizität (die zweite industrielle Revolution), diese Möglichkeit nahezu überall. Die technischen Entwicklungen stellen damit einen zweistufigen Veränderungsprozess dar. Die grundsätzliche Möglichkeit wird in der ersten Stufe geschaffen, die allgemeine Verfügbarkeit geht jedoch erst mit der zweiten Stufe einher. Diese allgemeine Verfügbarkeit führt anschließend zu massiven Auswirkungen auf Arbeit und Leben. Analog dazu wurden durch die Erfindung des Computers erstmals Prozesse informatorischer Arbeitstätigkeit durch Maschinen substituiert und durch die internetbasierte Vernetzung wird erneut eine allgemeine Zugänglichkeit geschaffen (Bubb 2006). Zweifelsohne führen das Internet und die Digitalisierung zu massiven Veränderungen in der Arbeitswelt und die Digitalisierung ist nach wie vor erst im Begriff, ihre Konsequenzen und ihr Potenzial zu entfalten. Handlungsbedarfe für die Neugestaltung von Arbeitssituationen wurden beispielsweise von Sträter und Bengler (2019) skizziert.

Ein weiterer Treiber für weitreichende Veränderungen in der Arbeitswelt sind krisenbedingte Umwälzungen. Beispielsweise löste der erste Weltkrieg (1914–1918) einen entscheidenden Schub in der Entwicklung von Frauenarbeit aus (Wellner 1981), da es kriegsbedingt zu einem Mangel an Arbeitskräften kam. Folglich wurden Frauen insbesondere in der Rüstungsindustrie akquiriert und es wurde in Assistenzsysteme bzw. technische Unterstützungssysteme investiert, um die schwere körperliche Arbeit für Frauen ausführbar zu gestalten (Wellner 1981). Auch die COVID-19 Pandemie ist eine solche Krise, die das Potenzial zu kurzfristigen, radikalen aber auch innovativen Veränderungen im Kontext der Arbeit hat.

Im Ergebnis der rasanten Verbreitung von COVID-19 und der damit verbundenen Folgen für die Gesundheit der gesamten Bevölkerung wurden durch die Bundesregierung im März 2020 weitreichende Hygienemaßnahmen und die Beschränkung sozialer bzw. physischer Kontakte beschlossen (Bundesregierung 2020a). Die bis heute in unterschiedlichem Umfang gültigen Maßnahmen sollen dabei die Verbreitung des Virus eindämmen sowie eine Verringerung seiner Reproduktionsrate bewirken. Nachdem die Maßnahmen, wie z. B. allgemeine Verhaltensregeln, Reduzierung der Kontakthäufigkeit, Gestaltung der Arbeitsumgebung sowie Hygiene- und Reinigungsmaßnahmen (Robelski et al. 2020), insbesondere auch im Kontext der Erwerbsarbeit umgesetzt werden müssen (Bundesregierung 2020a), kam es zu teilweise drastischen Auswirkungen auf Arbeitsformen und Arbeitsprozesse. Dieser Bewältigungsprozess könnte zu einem wertschöpfungsintegrierten Lernprozess geführt haben, da Unternehmen und Organisationen beispielsweise vernetztes und flexibles Arbeiten ermöglichten und bestehende Strukturen und Arbeitsabläufe adaptierten, um arbeitsfähig zu bleiben. Diesen Veränderungsprozess begleitete und erforschte das Projekt COVID19LL, um wissenschaftlich fundierte Aussagen darüber tätigen zu können, inwiefern und in welchem Maße Veränderungen in der Arbeitswelt erfolgreich und nachhaltig entstehen.

## Theoretischer Hintergrund

### Verlauf der Pandemie in Deutschland

Am 25. März 2020 reagierte der Deutsche Bundestag auf die Ausbreitung des Virus in Deutschland mit ersten Gesetzentwürfen zum Schutz der Bevölkerung, zur Entlastung der Krankenhäuser sowie zum Sozialschutz angesichts der Krise (Deutscher Bundestag 2020). Die Ministerpräsidentinnen und -präsidenten einigten sich zudem auf ein bundesweites Vorgehen bei der Einführung präventiver Maßnahmen inklusive der Einschränkung sozialer Kontakte (Bundesregierung 2020b). Erst nach mehr als einem Monat im sogenannten Lockdown stellten sich geringere Inzidenzzahlen ein, sodass es im Mai in einzelnen Bundeländern zu schrittweisen Lockerungen der Kontaktbeschränkungen kommen konnte (Bundesregierung 2020c). Im Sommer folgte eine Phase niedriger Inzidenzwerte, welche jedoch trotz der lokal fokussierten *Hotspotstrategie* im Spätherbst nicht aufrechterhalten werden konnte (Bundesregierung 2020d). Aus diesem Grund wurden am 02. November 2020 vom Bundestag erneute Einschränkungen sozialer Kontakte beschlossen (Bundesregierung 2020e). Dieser „Lockdown light“ konnte den exponentiellen Anstieg der Infektionszahlen jedoch nicht verhindern, weshalb es am 16. Dezember zu einem zweiten Lockdown kam (Bundesregierung 2020f). Die pandemische Lage wurde dabei zusätzlich durch das Auftreten neuartiger Virusvarianten verschärft (Bundesregierung 2021a). Die Ausbreitung der Alpha-Variante von SARS-CoV‑2 in Deutschland (Robert-Koch-Institut 2021) führte dazu, dass trotz sinkender Infektionszahlen im ersten Quartal 2021 der Lockdown vorerst aufrechterhalten wurde (Bundesregierung 2021b). Nach dem Rückgang der Inzidenzzahlen im Januar und Februar 2021 kam es ab Mitte März zu einem erneuten Anstieg der Infektionen (Bundesregierung 2021c). Vor diesem Hintergrund betonte Bundesgesundheitsminister Spahn, dass dieser Anstieg nicht allein durch die im Dezember 2020 angelaufene Impfkampagne zu stoppen sei und forderte eine konsequente Einhaltung und Durchsetzung der Infektionsschutzmaßnahmen (Bundesministerium für Gesundheit 2021). Erst im Mai 2021 kam es zu einem deutlichen Abfall der Inzidenzen, so dass ein Großteil der bestehenden Kontaktbeschränkungen schrittweise zurückgenommen werden konnte (Bundesregierung 2021d).

### Aktuelle Forschung zu Auswirkungen auf die Arbeitsweise und -formen

Die mit der Verbreitung des Virus einhergehenden Veränderungen in der Arbeitswelt sind derzeit Gegenstand zahlreicher Forschungsaktivitäten. So wurden im Rahmen einer Online-Befragung 211 Technologie‑, Digitalisierungs- und KI-Expertinnen und -Experten im April 2020 hinsichtlich möglicher Entwicklungspfade der langfristigen Veränderungen durch die COVID-19 Pandemie in der Arbeitswelt befragt. Dabei zeigte sich, dass 92 % der Teilnehmenden von einer Beschleunigung der digitalen Transformation ausgehen und 85 % den Fortbestand des Home-Office und virtueller Meetings auch nach der Krise sehen (Krcmar und Wintermann 2020). 44 % der Befragten erwarteten eine neue Führungskultur mit mehr Vertrauen und weniger Kontrolle (Krcmar und Wintermann 2020). Diese notwendige Entwicklung beschreibt auch Diewald (2020). Demzufolge sollten Absichten und Folgen in Bezug auf Home-Office transparent kommuniziert werden. „Eine solche Transparenz ist Voraussetzung dafür, dass (…) Vertrauen möglich wird, um den Kontrollverlust durch den Wegfall der Anwesenheitspflicht zu kompensieren“ (Diewald 2020, S. 39).

Kunze et al. (2020) fanden in ihrer Befragung von 700 Beschäftigten zu 9 Erhebungszeitpunkten tiefgreifende Veränderungen im Arbeitsalltag der Beschäftigten, bedingt durch die Pandemie. Die Flexibilisierung von Arbeitsort und Arbeitszeit führt demnach zu einem höheren Grad an Autonomie und Beschäftigte gaben eine verbesserte Arbeitszufriedenheit, Arbeitsleistung und Work-Life-Balance an. Negativ hervorgehoben wurden insbesondere Aspekte wie die soziale Isolation, Überarbeitung und emotionale Erschöpfung. Zahlreiche Beschäftigte wünschen sich für die Zukunft ein hybrides Arbeitsmodell mit einem Mix aus Home-Office und Präsenz (Kunze et al. 2020).

Ein ähnliches Bild zeigt sich bei einer repräsentativen Befragung der forsa Politik- und Sozialforschung GmbH (forsa 2020), bei der im Auftrag der DAK Bayern im November 2020 1006 abhängig Beschäftigte in Bayern zum Thema Home-Office befragt wurden. Dabei zeigte sich, dass 44 % der Beschäftigten auch nach der Pandemie einmal oder mehrmals in der Woche im Home-Office arbeiten möchten. Von 73 % der Befragten wurde angegeben, dass sie im Home-Office produktiver arbeiten können, jedoch benannten 63 % der Befragten eine stärkere Vermischung von Arbeits- und Privatleben als negative Konsequenz. Etwa 70 % der Beschäftigten gaben an, dass ihnen der soziale Austausch und der soziale Kontakt zu Kolleginnen und Kollegen fehlen und 33 % nannten das Fehlen der gewohnten Struktur in ihrem Arbeitsalltag als negative Auswirkung des Home-Office auf das eigene Wohlbefinden (forsa 2020).

Zusammengefasst zeigt sich in der Literatur ein starker Fokus auf die Themen Beschleunigung der digitalen Transformation (Krcmar und Wintermann 2020; Engels 2020; Soto-Acosta 2020; BMWi 2021), Veränderungen von Arbeitszeit (Flexibilisierung) und Arbeitsort (Home Office) (Kunze et al. 2020; forsa 2020; Hofmann et al. 2020; Grzech-Sukalo 2020) sowie ein Wandel der Führungskultur (Diewald 2020; Krcmar und Wintermann 2020; Umbs 2020; Sandrock et al. 2020; Hofmann et al. 2020; Ipsen et al. 2021).

### Forschungsziel

Im Rahmen dieses Forschungsvorhabens soll qualitativ ermittelt werden, welche pandemiebedingten Veränderungen im Kontext der Arbeit überregional und sektorenübergreifend auftreten, wie diese zu bewerten sind und welche neuen Arbeitsweisen sich auch in einer postpandemischen Zeit bewähren können. Die Forschungsfrage lautet somit: Welche Maßnahmen und Lösungen wurden von verschiedenen Branchen eingeführt und ggf. modifiziert/optimiert, um in der veränderten Situation durch COVID-19 arbeitsfähig zu sein? Aufbauend auf den Ergebnissen wird zudem diskutiert, welche Maßnahmen und Lösungen sich auf andere Branchen und in die Zeit nach COVID-19 übertragen lassen könnten. Im Folgenden wird das methodische Vorgehen zur Beantwortung der Forschungsfrage erläutert. (Für weiterführende Informationen zum theoretischen Hintergrund siehe Bengler et al. (2021)).

## Methodik der realisierten Erhebung

### Entwicklung eines Konzepts – Prozessmodell

**Figure 1:**
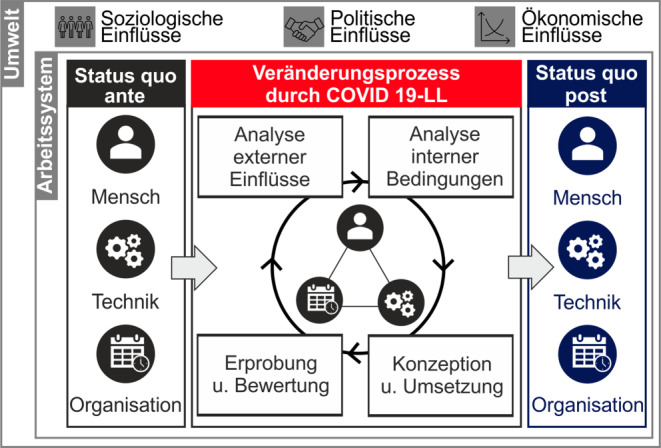

Als Bezugsbasis der qualitativen Erhebung wurde das Arbeitssystem gewählt, zu der unterschiedliche Modelle herangezogen werden können. Ein häufig genutzter Ansatz ist das MTO-Konzept (Strohm 1997), welches das Zusammenspiel von Mensch, Technik und Organisation betont und dabei die Arbeitstätigkeit in den Fokus stellt. Da im Rahmen des vorliegenden Forschungsvorhabens ein Veränderungsprozess betrachtet wird, wurde auf Basis des MTO-Konzepts ein Prozessmodell entwickelt, welches die systematische Betrachtung der Zeit vor der Pandemie, den dynamischen Veränderungsprozess während der Pandemie sowie einer zukünftigen Phase nach der Pandemie ermöglicht (Abb. 1).

Im Rahmen des adaptierten Modells liegt der Fokus zunächst auf der Erhebung des Status quo ante, wobei die drei Dimensionen Mensch, Technik und Organisation beleuchtet werden. Anschließend wird der Veränderungsprozess in Unternehmen und Organisationen über den Verlauf der Pandemie betrachtet. Bei dem pandemiebedingten Veränderungsprozess handelt es sich um einen dynamischen Vorgang, der durch zahlreiche interne und externe Faktoren (wie beispielsweise politische Maßnahmen, die Zu- bzw. Abnahme des Infektionsgeschehens oder die unternehmerischen Gegebenheiten) beeinflusst wird und mit wechselnden Anforderungen einhergeht. Das Modell bezieht zusätzlich allgemeine politische, soziologische und ökonomische Einflüsse mit ein, die während der Pandemie auf das Arbeitssystem wirken. Auch in der Betrachtung des Veränderungsprozesses werden die Dimensionen Mensch, Technik und Organisation analysiert. Die Betrachtung einer post-pandemischen Zeit kann zum aktuellen Zeitpunkt, in dem die Pandemie noch nicht überwunden ist, lediglich im Sinne einer Vision sowie einer durch die arbeitende Bevölkerung gewünschten Zukunft verstanden werden. Da das Modell jedoch Allgemeingültigkeit besitzen soll, wird der Status quo post zusätzlich als Referenz für die Bewertung des Veränderungsprozesses angesehen.

Auf Basis dieses Prozessmodells wurde der Leitfaden für die Datenerhebung in Form von teilstrukturierten Interviews entwickelt. Anhand der SPSS Methode zur Konstruktion von Interviewleitfäden wurden zunächst Fragen gesammelt, die anschließend mithilfe von Prüffragen revidiert wurden. In einem weiteren Schritt wurden die Fragen sortiert und zu übergeordneten Leit- und Teilfragen subsumiert (Helfferich 2011).

Der Aufbau des finalen Interviewleitfadens ist wie folgt:

1. Allgemeine Fragen zum Unternehmen und zur Person: Es wurde der Wirtschaftszweig nach WZ2008 (Statistisches Bundesamt 2008) erfragt sowie Umsatz und Anzahl der Beschäftigten (European Commission 2020). Die interviewte Person beschrieb die Tätigkeiten, Dienstleistung bzw. Produkte und die eigene Rolle im Unternehmen/der Organisation.
2. Status quo ante: Beschreibung des Arbeitssystems vor der Pandemie auf den Ebenen Mensch, Technik und Organisation zur Erfassung der ursprünglichen Rahmenbedingungen.
3. Veränderungsprozess: Fokussierung einzelner Maßnahmen, welche in den drei Dimensionen des MTO-Konzepts eingeführt wurden sowie der Entwicklungsprozess, dem die Maßnahmen unterlagen. Weiterhin wurden die internen Voraussetzungen sowie die externen Treiber für die Entwicklung und Adaption der Maßnahmen erfasst. Neben der Konzeption und Umsetzung der Maßnahmen wurde zudem eine Bewertung aus Perspektive der Befragten eingeholt, wie sich die Maßnahmen auf Wirtschaftlichkeit, Zusammenarbeit, Arbeitszufriedenheit, Arbeitssicherheit und Gesundheitsschutz, Einfluss und Gestaltungsmöglichkeit der beschäftigten, Vereinbarkeit von Arbeit und Leben sowie ökologische Nachhaltigkeit auswirkten.
4. Status quo post: Abschließend wurden die angesprochenen Maßnahmen hinsichtlich ihrer Transferpotenziale in eine Zeit nach der Pandemie bewertet und zusätzlicher Handlungsbedarf identifiziert.

### Empirische Erhebung – Interviews

Die Datenerhebung wurde überregional in den drei Bundesländern Bayern, Nordrhein-Westfalen und Sachen durchgeführt. In einer Pilotphase der empirischen Erhebung wurde die Schwerpunktsetzung für den entwickelten Interviewleitfaden abgesichert. Zu diesem Zweck wurden acht Interviews mit Arbeitgeber- und Arbeitnehmervertretungen geführt. In diesen Gesprächen wurde zum einen überprüft, ob sich alle von den Befragten als relevant eingeschätzten Aspekte durch das entwickelte Prozessmodell abbilden lassen, und zum anderen wurde die Auswahl der zu berücksichtigenden Branchen für die Interviews mit Unternehmensvertretungen komplettiert.

Basierend auf dieser Vorarbeit wurden in der Hauptphase der empirischen Erhebung insgesamt 52 Interviews mit Vertretungen aus 34 unterschiedlichen Unternehmen und Organisationen durchgeführt. An den Interviews nahmen 16 Frauen und 36 Männer teil, von denen insgesamt 35 (67 %) eine Führungsposition innehatten. Die Aufteilung der Vertretungen nach Unternehmensgröße und Wirtschaftszweig des Unternehmens bzw. der Organisation kann Tab. 1 und 2 entnommen werden. Einzelhandel und Gastronomie wurden von der Datenerhebung ausgeschlossen, da sie während der Lockdowns ihre eigentliche Dienstleistung nicht erbringen konnten. Es fanden Interviews mit Vertretungen aus allen drei Wirtschaftssektoren statt, mit Schwerpunkten auf dem sekundären (Industrieller Sektor) und dem tertiären Sektor (Dienstleistungssektor). Das erste Interview in einem Unternehmen wurde jeweils mit einem Mitglied der Geschäftsführung, einer Bereichsleiterin oder einem Bereichsleiter bzw. einer entsprechenden Führungskraft durchgeführt. Konnten in diesem ersten Interview besonders vielversprechende Maßnahmen und Ansätze identifiziert werden, wurden weitere Interviews mit Mitarbeitenden geführt, die entweder an der Maßnahmenentwicklung und -umsetzung beteiligt waren oder die in ihren Arbeitsabläufen unmittelbar durch die umgesetzten Maßnahmen beeinflusst wurden.

**Table 1:** 

Wirtschaftszweig	U	I
Verarbeitendes Gewerbe	10	19
Land- und Forstwirtschaft	6	6
Öffentliche Verwaltung; Sozialversicherung	5	7
Gesundheits- und Sozialwesen	4	7
Information und Kommunikation	3	7
Erziehung und Unterricht	2	2
Erbringung von freiberuflichen, wissenschaftlichen und technischen Dienstleistungen	1	1
Erbringung sonstiger Dienstleistungen	1	1
Handel	1	1
Kunst, Unterhaltung und Erholung	1	1

**Table 2:** 

Unternehmensgröße	U	I
Große Unternehmen	10	20
Mittlere Unternehmen	6	9
Kleine Unternehmen	5	5
Kleinstunternehmen	3	3
Organisationen	10	15

Die Durchführung der Interviews erfolgte zwischen Oktober 2020 und Mai 2021 und damit kurz vor bzw. während des zweiten bundesweiten Lockdowns. Dementsprechend konnten die Gespräche nicht in Präsenz geführt werden, sondern wurden online über Videokonferenztools durchgeführt. Von den Interviews wurden Audioaufzeichnungen angefertigt, welche anschließend anhand des Regelsystems nach Dresing und Pehl (2015) vollständig transkribiert wurden. Die Interviewtranskripte wurden mittels der Software MAXQDA 2020 (VERBI Software 2019) codiert. Bei der Entwicklung des Codierungsschemas wurden induktives und deduktives Vorgehen miteinander verknüpft (Mayring 2010). Die Kategorienbildung, für die in den Unternehmen entwickelten und umgesetzten Maßnahmen, wurde induktiv aus dem gesammelten Material abgeleitet. Dieses Vorgehen wurde durch eine zweite, auf dem entwickelten Prozessmodell beruhende Codierungsebene ergänzt. Dementsprechend wurden für die identifizierten Maßnahmen und abgeleiteten Maßnahmengruppen jeweils die einzelnen Stufen des Prozessmodells aus einer MTO-Perspektive herausgearbeitet.

### Aufbereitung der Ergebnisse – narrative Szenarien

Für die Aufbereitung der Ergebnisse wurde die Szenariotechnik gewählt. Diese Methodenwahl basiert auf folgenden Charakteristika des Forschungsvorhabens:

1. Die Szenariotechnik ermöglicht die Einbindung von Fachkundigen, wobei die Unternehmensvertretungen als Expertinnen bzw. Experten in eigener Sache verstanden werden. Einerseits werden Informationen der Befragten durch die Szenariotechnik synthetisiert und damit umfassendes interdisziplinäres Systemwissen erzeugt. Andererseits dienen die Szenarien als Kommunikationsmittel zwischen Akteuren verschiedener Sphären (Kosow und León 2015)
2. In der Zielbildungsfunktion der Szenarien werden zukunftsrelevante Lösungen hinsichtlich ihrer künftigen Umsetzung im Unternehmen geprüft. Es werden normativ-narrative Szenarien entwickelt, die eine mögliche und wünschbare Zukunft konkret vorstellbar machen und damit eine erweiterte Basis für Diskussionen um Handlungsoptionen bieten (Kosow und Gaßner 2008). Im konkreten Fall wird dies durch die Formulierung der vorgefundenen Situation in Best-Practice-Szenarien realisiert, die auf einer konkreten Zielvorstellung aufgebaut werden.
3. Da das Projekt auf verhältnismäßig wenigen, qualitativen Datensätzen aufbaut, werden für die praxisorientierte Form der Ergebnisaufbereitung narrative Szenarien gewählt (Kosow und Gaßner 2008). Diese ermöglichen es in erster Linie, die vorgefundenen komplexen Situationen plausibel und verständlich darzustellen und daraus Konsequenzen nachvollziehbar abzuleiten. Diese Aufbereitungsform erleichtert auch die Erreichbarkeit der Adressaten, im vorliegenden Fall betriebliche Praktiker, die Anregungen und erfolgsversprechende Lösungen aufnehmen und im eigenen Kontext umsetzen sollen.

Ein normativ-narrativer Ansatz, wie für die Ergebnisdarstellung ausgewählt, wird primär quasi-literarisch gestaltet, in Form kurzer Erzählungen über fiktive Personen und Institutionen. Diese Darstellungsform fordert ein hohes Maß an Detaillierung, Konkretheit und Realismus. Das Szenario wird als glaubwürdig eingestuft, wenn die erzählerische Gestaltung eine ausreichende Konsistenz und Plausibilität besitzt (Steinmüller 1997; Kosow und Gaßner 2008). Die Hauptfunktion narrativer Szenarien ist die Bildung eines fundierten Verständnisses der Einflussfaktoren und Wechselwirkungen zwischen einzelnen Faktoren sowie Kenntnisse über die Handlungsspielräume, in denen die Maßnahmen getätigt werden.

Voraussetzung für die Formulierung der Szenarien war es, die erfassten Informationen der Interviews in ihrer Breite zu durchdringen und auf Gemeinsamkeiten und Unterschiede zu prüfen. Dazu wurden zunächst aus den kodierten Interviews alle relevanten Maßnahmen extrahiert. Die Schlüsselfaktoren der Szenarien werden durch die im Prozessmodell abgebildeten und in den Interviews erfassten Einflussfaktoren repräsentiert. In einem zweiten Schritt wurden die identifizierten Maßnahmen zur Bewältigung der Corona-Pandemie samt zugehörigen Erfolgsfaktoren durch die Projektbearbeitenden geclustert. Dabei zeigten sich viele ähnliche Maßnahmen verteilt über alle Branchen- bzw. Wirtschaftssektoren, weshalb für die weitere Auswertung die identifizierten Maßnahmencluster in den Mittelpunkt gestellt wurden.

## Szenarien

Durch den prozessmodellbasierten Ansatz einer möglichst umfassenden Beschreibung der Veränderungen in den befragten Unternehmen und Organisationen, wurde eine Vielzahl von kleinen, jedoch miteinander verknüpften Maßnahmen identifiziert. Diese können zur Systematisierung den folgenden Wirkbereichen zugeordnet werden:

- Anpassung von internen und externen Kommunikationsstrukturen
- Flexibilisierung der Arbeitsorganisation
- Flexibilisierung der Arbeitszeit
- Flexibilisierung des Arbeitsorts
- Hygienekonzepte
- Maßnahmen zum operativen Umgang mit Krisensituationen
- Modifikation von Geschäftsprozessen (insb. durch Digitalisierung)

Maßnahmen dieser Wirkbereiche sind in allen drei Wirtschaftssektoren zu finden. Der maßgebliche Unterschied zwischen den Maßnahmen besteht im Bedarf der physischen Anwesenheit und physischer Kontakte am Arbeitsplatz zur Ausübung der primären Arbeitstätigkeit. Aus diesem Grund wurden die Ergebnisse in drei Szenarien komprimiert, die sich in der Art der Arbeitsausführung unterscheiden in:

- Arbeit in Bereichen, in denen physische Anwesenheit unabdingbar ist und keine Möglichkeit besteht, aus der Ferne zu arbeiten
- Arbeit in Bereichen, in denen teilweise physische Anwesenheit erforderlich ist und in manchen Bereichen Arbeiten aus der Ferne möglich ist
- Arbeit in Bereichen, in denen keine physische Anwesenheit erforderlich ist, so dass die Arbeit vollständig aus der Ferne erledigt werden kann

Die auf die Arbeitsausführung ausgerichteten Szenarien werden durch ein viertes Szenario ergänzt, welches Vorgehen und Erfahrungen der befragten Unternehmen und Organisationen im operativen Umgang mit krisenbedingten Herausforderungen bzw. der Pandemie, inklusive der Entwicklungs- und Umsetzungsprozesse von neuen Maßnahmen umfasst.

Gemäß den Grundsätzen für die Szenariengenerierung wird eine übergeordnete Zielsetzung für alle formulierten Szenarien konzipiert: Ziel des Unternehmens/der Organisation ist es, die Arbeitsfähigkeit in Anbetracht der pandemiebedingten Herausforderungen aufrechtzuerhalten bzw. zu fördern und dabei die Standards *Gute Arbeit* einzuhalten.

Für jedes Szenario wird in Form der spezifischen Zielstellung und der Unternehmens‑/Organisationsbezüge der Geltungsbereich des Szenarios definiert. Die dargestellten Szenarien sind allgemeingültig gehalten und explizit nicht einzelnen Wirtschaftssektoren und nur beispielhaft einzelnen Branchen zugeordnet, damit sie leicht in andere Bereiche transferiert werden können. Die Szenarien können im Detail unter folgendem Link eingesehen werden: https://osf.io/gxm8j/.

### Szenario „Ortsgebundenes Arbeiten“

Das erste Szenario adressiert ortsgebundenes Arbeiten. Relevant ist dieses Szenario beispielsweise für produzierende Unternehmen, ambulante oder stationäre Pflege sowie Anbau bzw. Ernte land-/oder forstwirtschaftlicher Erzeugnisse. Physische Anwesenheit ist in diesem Szenario unabdingbar, was sich beispielsweise aus dem für die Erbringung einer Dienstleistung zwingenden persönlichen Kontakt ergibt oder aus der technologischen Notwendigkeit.

In diesem Szenario durchlebt eine fiktive Person einen Arbeitstag im Unternehmen vor Ort. Dabei werden insbesondere Aspekte des Hygienekonzepts, die räumliche Trennung von Personen und Arbeitsbereichen und die Möglichkeit zu Weiterbildung und Schulungen dargestellt. Einen zentralen Baustein des Szenarios stellen die Kommunikation des Unternehmensstatus sowie die Kommunikation der Etablierung von Maßnahmen dar.

Der Logik des MTO-Ansatzes entsprechend, werden die Inhalte im Folgenden differenziert, die im Rahmen des Szenarios zu ortsgebundenem Arbeiten adressiert werden.

#### Mensch

Ein Themenkomplex, der im Szenario zu ortsgebundenem Arbeiten von großer Bedeutung ist und der Dimension Mensch zugeordnet wird, dreht sich um die Themen Interesse und Bedürfnisse. In den Interviews wurde die Möglichkeit zur **Mitsprache** der Belegschaft häufig hervorgehoben, da es pandemiebedingt vermehrt zu top-down Entscheidungen kam. Im Sinne der Belegschaft sollten Möglichkeiten zur Mitsprache in ausgewählten Bereichen jedoch auch während der Pandemie gewährt werden. So wünschen sich viele Beschäftigte mehr Flexibilität und Mitsprache bei der Planung von Schichtarbeit und Arbeitszeit.

Von den Teilnehmenden wurde zudem der fehlende bzw. reduzierte** soziale Kontakt **angesprochen. Der Wegfall gemeinsamer Pausen und Treffen vor Ort beeinflussten das Wir-Gefühl und den informellen Austausch zwischen Beschäftigten. Anhand gezielter Maßnahmen und Angebote kann der Austausch in der Belegschaft gefördert werden.

Die **Qualifikation** und **digitale Kompetenz** der Belegschaft ist ein weiterer Baustein des Szenarios. Vereinzelt kam es in Unternehmen pandemiebedingt zu Kurzarbeit oder der Substitution auszuführender Tätigkeiten. Die Zeit für zwischenzeitlich nicht ausführbare Tätigkeiten hätte nach Einschätzung eines Interviewpartners gewinnbringend für Weiterbildungen und Schulungen genutzt werden können.

**Persönliche Schutzausrüstung**, insbesondere das Tragen der Masken (FFP2-Masken, medizinische Masken oder Mund-Nase-Bedeckungen), welche während der Pandemie bei ortsgebundenem Arbeiten in Unternehmen und Organisationen obligatorisch war, wurde in den Interviews zwar als Belastung beschrieben, jedoch gleichzeitig überwiegend positiv bewertet, da nur so das Arbeiten vor Ort möglich ist. Dass ausreichend Schutzausrüstung von Unternehmen/Organisationen zur Verfügung gestellt werden und die Masken am Arbeitsplatz abgenommen werden können, erhöht die Akzeptanz in der Belegschaft.

Die Einführung eines Standardprozesses bei Verdachtsfällen wurde im Rahmen der Interviews als wertvoller Beitrag für die Vermittlung eines **Sicherheitsgefühls** für die Beschäftigten beschrieben. Insbesondere um Unsicherheiten und Ängste der Beschäftigten vorzubeugen, sind Infektionsfälle umgehend zu melden.

#### Technik

In der Dimension Technik lässt sich die Einführung und der Einsatz von **Informations- und Kommunikationstechnologien** verorten. Die Bereitstellung von Information über synchrone und asynchrone Wege, um einerseits die Möglichkeit zu geben, Fragen zu stellen und andererseits sicherzustellen, dass die gesamte Belegschaft jederzeit auf die Informationen zugreifen kann, wurde im Rahmen der Interviews hervorgehoben. Ebenso wurde auf positive Resultate durch die Einführung von Online-Bewerbungsgesprächen und Online-Terminvereinbarung zur Aufrechterhaltung von Prozessen verwiesen.

Hinsichtlich der **Arbeitsmittel** wurden vorwiegend Hygieneaspekte aufgeführt. Dies umfasst neben der Einführung von Plexiglasscheiben zwischen Arbeitsplätzen auch die Desinfektion der Räumlichkeiten und die Möglichkeit persönlicher Desinfektion. Sowohl mit Blick auf die persönliche Schutzausrüstung als auch auf die Arbeitsmittel gingen die Befragten teilweise davon aus, dass angepasste Hygienekonzepte auch nach der Pandemie bestehen bleiben, da sich die Maßnahmen scheinbar auch positiv auf die Prävention anderer Erkrankungen, wie beispielsweise grippale Infekte, ausgewirkt haben. Im Vergleich zu den Vorjahren konnte in manchen Unternehmen ein Rückgang der AU-Tage verzeichnet werden.

#### Organisation

Die Dimension Organisation umfasst vorwiegend Aspekte zu **räumlichen Bedingungen** und **Arbeitsort bzw. -zeit**. Neben der Schichtzusammensetzung aus festen, kleinen Teams führten auch zeitlich versetzte Pausen dazu, dass weniger Kontakte zwischen Beschäftigten verschiedener Teams entstehen. Dies ermöglicht einerseits, dass die Arbeit auch im Falle eines Infektionsfalls Großteils weitergeführt werden kann. Andererseits kann es zu einer Spaltung in Arbeitsbereichen führen, da der Personenkreis, der sich vor Ort regelmäßig sieht/begegnet, stark eingeschränkt ist.

Die **Kommunikationsstrukturen und -formen** zur Verbreitung von **Entscheidungen** stellen in allen Szenarien einen wichtigen Anteil dar. In den Interviews wurden die Notwendigkeit der Begründung und der nachvollziehbaren Erläuterung eingeführter Maßnahmen sowie deren Anpassung betont. Insbesondere die frühzeitige Einbeziehung des Betriebsrats kann dabei einen positiven Beitrag leisten. Die regelmäßige und transparente Kommunikation von Neuerungen führt den Interviews zufolge zu einer erhöhten Akzeptanz in der Belegschaft.

### Szenario „Teilweise ortsgebundenes Arbeiten“

Das zweite Szenario adressiert teilweise-ortsgebundenes Arbeiten. Es deckt ein breites Spektrum von Arbeitstätigkeiten sowohl im Sekundär- als auch im Tertiärsektor ab, wobei Sachbearbeitung, Geschäftsführung und Verwaltung beispielhafte Anwendungsfälle darstellen. Definiert wird das Szenario dadurch, dass die Leistungserbringung des Unternehmens/der Organisation teilweise physische Anwesenheit erfordert, wie z. B. für Abstimmungsprozesse und Unterschriften. Die Umsetzung vieler Aufgabenschritte ist jedoch nicht ortsgebunden und kann auch aus der Ferne erfolgen.

Dieses Szenario begleitet eine fiktive Person an einem Arbeitstag, welcher durch einen Wechsel seines Arbeitsplatzes zwischen Büro und Home-Office geprägt ist. Im Fokus des Szenarios stehen die flexible Wahl des Arbeitsortes in Abhängigkeit der Arbeitsaufgaben, die daraus resultierenden Anforderungen und Anpassungen der internen und externen Kommunikationsprozesse sowie die Auswirkungen auf die Vereinbarkeit von Arbeit und Privatleben.

#### Mensch

Der Dimension Mensch sind insbesondere Aspekte zuzuordnen, in denen sich die **flexible Wahl des Arbeitsortes auf die Arbeitsweise** und die Integration des Arbeitstages in den Alltag der Mitarbeitenden auswirkt. In Bezug auf die eigentliche Arbeitstätigkeit ist hier die unterschiedliche Eignung von Arbeitsorten für verschiedene Aufgaben zu nennen. Während insbesondere kollaborative Zusammenarbeit und Entscheidungsfindungsprozesse bei komplexen und kritischen Themen von Abstimmungen in Präsenz profitieren, sind Aufgaben, welche in konzentrierter Einzelarbeit zu bearbeiten sind, gut für Arbeitsphasen von Zuhause geeignet, sofern dort die notwendigen Arbeitsbedingungen geschaffen werden können.

Die Arbeitsphasen in Präsenz tragen ebenfalls dazu bei, die **sozialen Kontakte** und den **Austausch zwischen den Mitarbeitenden** aufrecht zu erhalten, wodurch sowohl die abteilungsübergreifende Zusammenarbeit und Ausnutzung von Synergiepotentialen innerhalb des Unternehmens gefördert werden kann als auch ein wichtiger Beitrag für das Wohlbefinden der Beschäftigten durch sozialen Anschluss geleistet wird. Die Vereinbarkeit von Arbeit und Privatleben kann verbessert werden, wenn die Flexibilisierung des Arbeitsortes durch flexible Arbeitszeitregelungen ergänzt wird und die Reduktion der Arbeitszeit vor Ort auch die Reduktion der Anzahl an Dienstreisen einschließt.

Um von den Vorteilen einer Flexibilisierung des Arbeitsortes bestmöglich zu profitieren, spielt die konkrete Ausgestaltung innerhalb des Unternehmens eine wichtige Rolle. Offenkundig ist, dass je nach Tätigkeitspektrum innerhalb des Unternehmensbereichs, den Kompetenzen und der Arbeitsweise der beteiligten Mitarbeitenden und Führungskräfte sowie den zur Verfügung stehenden Arbeitsbedingungen im Home-Office, die Regelungen zur **Wahl des Arbeitsortes kontextabhängig gestaltet** werden sollten. Abseits bestehender Absprachen kann die Möglichkeit zu deren flexibler Anpassung im konkreten Bedarfsfall sowohl die Effektivität der Arbeitsprozesse als auch das Erfüllen privater Verpflichtungen begünstigen.

In der beschriebenen Form stellen Flexibilität und **Mitsprache bei der Wahl des Arbeitsortes** ein vielfach gewünschtes Zukunftskonzept dar. Dabei bietet der Wechsel zwischen Präsenz und Homeoffice ein großes Potenzial, um nicht nur die Vorteile für die Abstimmung von Arbeit und Privatleben zu ermöglichen, sondern zugleich zentrale Probleme reiner Home-Office-Lösungen, wie die Verringerung sozialer Kontakte in der Belegschaft, zu vermeiden.

#### Technik

In diesem Szenario stehen die Einführung oder vermehrte Anwendung von **Kommunikations- und Informationstechnologien** im Zentrum der Entwicklungen auf der Dimension Technik. Der verstärkte Fokus auf räumlich getrenntes Arbeiten erhöht den Bedarf für technische Lösungen, um formelle und informelle Kommunikationswege aufrecht zu erhalten. Besonders begünstigt werden die kommunikativen Abläufe, wenn verschiedene Interaktionsformen, wie Chat, Videokonferenz und Datenaustausch, in einer Software-Lösung integriert werden, um die Übersichtlichkeit der digitalen Kommunikation zu wahren. Zudem stellt die Möglichkeit Präsenzmeetings per Videokonferenz zu erweitern und so für Personen, die nicht in Präsenz arbeiten, zugänglich zu machen, eine wichtige Voraussetzung dafür dar, dass die Flexibilisierung des Arbeitsortes tatsächlich gelebt werden kann.

Ein weiterer Anwendungsfall für digitale Technologien zur Unterstützung des örtlich flexiblen Arbeitens ist die Anwesenheitsplanung. In diesem Szenario, in dem Mitarbeitende nur einen Teil ihrer Arbeitszeit in Präsenz arbeiten, sollten diese Phase auch gezielt für Arbeitsschritte genutzt werden, welche von der Anwesenheit vor Ort profitieren. Da hierzu insbesondere Abstimmungsprozesse zwischen Kolleginnen und Kollegen gehören, kann über eine Anwesenheitsplanung sichergestellt werden, dass die entsprechenden Personen vor Ort sind und Rücksprachen einfach möglich sind. Insgesamt stellt die Sicherstellung von **Transparenz über Anwesenheit, Erreichbarkeit** und **erbrachte Leistung** einen zentralen Erfolgsfaktor für flexible Arbeitskonzepte dar.

Neben den Abstimmungsprozessen innerhalb eines Unternehmens bietet der vermehrte Einsatz digitaler Kommunikationskanäle auch Möglichkeiten **Prozesse mit Kundenunternehmen zu vereinfachen**. So kann bspw. durch die Digitalisierung von Supportprozessen bis hin zur Fernwartung die Reaktionszeit und ggf. die Anzahl erforderlicher Dienstreisen reduziert werden. Die Ausweitung digitaler Prozesse in den Arbeitsabläufen des Unternehmens erhöht dabei die Anforderungen an eine umfassende IT-Ausstattung der Beschäftigten sowie effektive Datenschutzkonzepte.

#### Organisation

Auch wenn eine Feinabstimmung der Regelungen zum Arbeitsort an die individuellen Bedürfnisse von Mitarbeitenden wünschenswert ist, so ergeben sich auch auf der Organisationsebene relevante Maßnahmen. Zum einen sollten grundlegende Richtlinien zur Umsetzung der Arbeitsplatzwahl formuliert werden, auf die in den **Arbeitsverträgen** der Beschäftigten Bezug genommen werden kann. Des Weiteren ist zu erwarten, dass sich hierdurch der Anteil von Arbeitstätigkeiten, welche in Präsenz ausgeführt werden, verändert. Dementsprechend wird es für Unternehmen relevant, bestehende Raumkonzepte nicht nur hinsichtlich der Größe, sondern auch hinsichtlich der Ausstattung zu überdenken.

Die Flexibilisierung des Arbeitsortes ging in vielen der befragten Unternehmen auch mit einer **flexibleren Gestaltung der Arbeitszeitregelungen** einher. Während durch diese Maßnahme die Vereinbarkeit von Arbeit und Privatleben der Mitarbeitenden weiter gefördert wird, entstehen auch zusätzliche Anforderungen an die Arbeitsorganisation. Insbesondere für die Transparenz und Steuerung der Erreichbarkeit der Mitarbeitenden müssen einfache Lösungen gefunden werden. Neben einer generellen Flexibilisierung der Arbeitszeit haben sich während der Pandemie ergänzende Freiräume für Beschäftigte im Bedarfsfall als wertvoll erwiesen. So könnten während der Pandemie ermöglichte Karenztage oder Ausnahmeregelungen für das Arbeitszeitkonto Beschäftigten mit akuten privaten Verpflichtungen, wie dem Pflegen von Angehörigen oder dem Betreuen von Kindern, benötigte Spielräume in ihrer Arbeitsgestaltung schaffen.

### Szenario „Ortsungebundenes Arbeiten“

Im dritten Szenario wird das ortsungebundene Arbeiten dargestellt, womit beispielsweise Arbeitstätigkeiten aus den Bereichen IT-Dienstleistung, Entwicklung/Programmierung, Journalismus/Schriftstellerei, Social-Media-Marketing, telefonischer Kundensupport, Beratungstätigkeit, kommunale Verwaltung, Konstruktions- und Entwicklungsabteilung oder Personalabteilung abgedeckt werden. Eine Leistungserbringung erfordert demnach keine physische Anwesenheit und die Arbeitstätigkeiten können gänzlich aus der Ferne erledigt werden.

Im Szenario zu ortsungebundenem Arbeiten wird eine fiktive Person bei ihrer Arbeitstätigkeit begleitet, die erst seit Kurzem im Unternehmen arbeitet. Die Person arbeitet komplett von zu Hause aus, weshalb sich dieses Szenario vorwiegend auf die Vor- und Nachteile der Arbeit im Home-Office bezieht sowie auf die technologischen Möglichkeiten zur Arbeit über Distanz.

#### Mensch

In der Dimension Mensch sind zunächst Aspekte zu verorten, die den **persönlichen Ressourcen** zugeordnet werden können. Wenn die Arbeit ausschließlich von zu Hause aus mittels digitaler Technologien ausgeführt wird, kommt es für viele Personen zu einer verstärkten Entgrenzung von Arbeit und Privatleben. Durch den Wegfall von Wegezeiten und die enge Taktung von Online-Meetings kommt es für einige Beschäftigte zwar zunächst zu erhöhter Produktivität, jedoch geht diese Entwicklung auch mit einer zunehmenden Verdichtung von Arbeit einher. Dies verschärft sich zusätzlich dadurch, dass der soziale Anreiz für Pausen mit Kolleginnen und Kollegen entfällt und deshalb häufig kaum oder keine Pausen gemacht werden. Hinzu kommt das Gefühl vieler Beschäftigter ständig erreichbar sein und mehr leisten zu müssen, um zu zeigen, dass sie auch im Home-Office produktiv sind.

Die Situation im Home-Office impliziert ein hohes Maß an Eigenverantwortung und erfordert selbstorganisiertes und selbstständiges Arbeiten. Auf diese **Kompetenzen** sollte neben dem Aufbau digitaler Kompetenzen besonderes Augenmerk gelegt werden. Die offene Kommunikation von Erwartungen und die frühzeitige Kommunikation wichtiger Termine, inklusive einer gewissen Planungssicherheit kann der Belegschaft darüber hinaus helfen, die Arbeit in einem reinen remote Szenario besser zu bewältigen.

**Emotionale Bindungen** stellen einen weiteren zentralen Aspekt der Dimension Mensch im Szenario des ortsungebundenen Arbeitens dar. Aufgrund der pandemiebedingten gezielten Reduzierung persönlicher Kontakte hat, den Interviewergebnissen zufolge, die emotionale Bindung in der Belegschaft nachgelassen. Vertrauen sowie das Gefühl von Zusammengehörigkeit und Verbundenheit bauen sich über digitale Kommunikationstechnologien demnach langsamer auf und auch wenn die gemeinsame Bewältigung einer schwierigen Situation das Wir-Gefühl stärken kann, sollte ein gezielter Fokus auf den Zusammenhalt in Teams gelegt werden.

Dies kann beispielsweise durch dedizierte Treffen zur **Förderung des persönlichen Austauschs** in Präsenz mit einzelnen ausgewählten Personen gefördert werden. Auch die Einführung eines Mentoring-Programms, vor allem für neue Beschäftigte, sowie die Begünstigung eines regelmäßigen Austauschs kann den Zusammenhalt im Team fördern. Die gemeinsame Erarbeitung einer Netiquette, inklusive der potenziellen Übereinkunft zur Aktivierung von Webcams in Online-Meetings, kann dabei helfen auch non-verbale Reaktionen zu erfassen und persönliche Bedürfnisse zu berücksichtigen.

#### Technik

Die Dimension Technik umfasst in diesem Szenario vorwiegend Aspekte **digitaler Technologien und Prozesse**. Gerade für neue Beschäftigte ist der Einstieg ins Unternehmen bei rein virtueller/ortsungebundener Zusammenarbeit schwierig. Neben der Durchführung von Online-Vorstellungsgesprächen können virtuelle Unternehmensführungen einen guten Einblick ins Unternehmen geben. Ein formalisierter On-Boarding Prozess kann auch online durchgeführt werden und wenn die Arbeitsmaterialien per Post zugestellt werden, kann eine Remote-Einarbeitung, idealerweise mit der Unterstützung durch die IT-Abteilung, gelingen.

Die **Digitalisierung **von (Verwaltungs‑)Prozessen und die Einführung digitaler Signaturen setzen ein hohes Maß an Datenschutz voraus, stellen jedoch eine zwingende Voraussetzung für reine Remote-Szenarien dar.

Zudem sollten auch Kommunikationstechnologien für den formellen und informellen Austausch zur Verfügung stehen, was unter anderem die Einführung eines Gruppenchats, virtuelle Kaffeepausen oder Online-Teambuilding Angebote umfassen kann.

#### Organisation

Der Dimension Organisation werden im Rahmen des Szenarios zu ortsungebundenem Arbeiten Aspekte die **Führung** betreffend zugeordnet. Dies umfasst die nötige Sensibilisierung von Führungskräften im Hinblick auf die Führung auf Distanz. Es fällt vielen Führungskräften schwer einzuschätzen, wie es um den Arbeitsfortschritt und die Arbeitslast der Beschäftigten bestellt ist. Auch die Kommunikation komplexer Sachverhalte ist online schwerer zu vermitteln und die Durchführung von Besprechungen außerhalb der Kernarbeitszeit stellt für viele Beschäftigte im Home-Office ein Problem dar. Der Umgang mit diesen und vergleichbaren Themen sollte daher von Führungskräften entsprechend berücksichtigt werden.

Die **Flexibilisierung** von Arbeitszeit und Arbeitsort ist für viele Beschäftigte eine Chance zu einer effektiveren Zeitnutzung während der Arbeit. Das hohe Maß an Flexibilität und Gestaltungsfreiheit wird insgesamt positiv bewertet, ebenso die Tatsache, dass Arbeitsort und Wohnort durch die Möglichkeiten der digitalen Zusammenarbeit nicht zwangsläufig aneinandergebunden sind und der Wohnort nicht gleich dem Arbeitsort sein muss. Die standortübergreifende Zusammenarbeit hat sich durch die vermehrte Nutzung digitaler Technologien verbessert, die Kollaboration insbesondere in Kreativprozessen gestaltet sich jedoch schwieriger.

Der Aufbau neuer **Kundenkontakte** über reine Online-Formate sowie der **Netzwerkaustausch** stellt eine größere Herausforderung dar, jedoch wurde positiv hervorgehoben, dass Online-Formate zusätzliche Möglichkeiten bieten, Inhalte adressatengerecht zu präsentieren bzw. aufzubereiten.

Abschließend werden der Dimension Organisation Anpassungen von **Arbeitsverträgen** und **rechtlichen Rahmenbedingungen** zugeordnet. Die vertragliche Zusicherung eines erhöhten Maßes an Flexibilität hinsichtlich Arbeitsort und Arbeitszeit wurde von Führungskräften genannt, wobei es die Vorgaben zum Arbeitsschutz einzuhalten gilt.

### Operative Bewältigung

Dieses Szenario greift den im Prozessmodell adressierten Aspekt des Veränderungsprozesses als Reaktion auf die Pandemie sowie dabei relevante Erfolgsfaktoren und Hemmnisse auf. Die relevanten Aspekte sind überwiegend der Dimension Organisation des MTO-Ansatzes zuzuordnen, daher wird in der folgenden Darstellung auf eine Differenzierung nach den Aspekten Mensch, Technik und Organisation verzichtet.

Die Unternehmen und Organisationen standen mit dem schnellen Überschwappen der Pandemie nach Deutschland vor der Herausforderung, ihr Leistungsportfolio und damit auch die Organisation ihrer Wertschöpfung an die resultierenden Bedingungen anzupassen. Diese waren zu Beginn der Pandemie durch die Festlegung des weitgehenden Lockdowns geprägt. Die entscheidenden externen Einflussfaktoren für die Unternehmen und Institutionen waren über den gesamten Zeitraum die umzusetzenden Hygieneverordnungen und die Schließung von Kindertagesstätten und Schulen, die insbesondere Auswirkungen auf die verfügbare Personalkapazität hatte. Vor diesem Hintergrund wurde das Leistungs- und Serviceangebot der Unternehmen und Organisationen durch die Geschäftsleitungen hinterfragt und auf eventuell notwendige Leistungseinschränkungen oder Leistungsabsagen zur Beschränkung der persönlichen Kontakte geprüft.

Die Unternehmen und Organisationen reagierten auf die Situation mit **Leistungsabsagen**, wodurch unter Umständen jedoch neue Aufgaben in Form der Rückabwicklung entstanden, mit Einschränkung aller nicht zwingend notwendigen Leistungen, die aufgrund der Hygieneverordnungen nicht oder nur unter erhöhtem Aufwand möglich waren sowie mit **Umstellung auf kontaktlose Leistungserbringung bzw. Alternativangebote**. Damit waren in unterschiedlichem Umfang, aber in allen Bereichen zusätzliche Aufwände bei der Leistungserbringung erforderlich.

Wenig Relevanz hatte, zumindest im Untersuchungszeitraum, die Problematik **unterbrochener Lieferketten** für die Unternehmen. Zwar waren Materiallieferungen und Auslieferungen mit höherem Aufwand verbunden, wurden jedoch als beherrschbar eingeschätzt. Die mit Leistungsabsagen u. U. verbundenen Produktionsstillstände konnten durch die **Kurzarbeitsregelung** abgefedert werden. Teilweise wurden Beschäftigte anderen Geschäftsbereichen und Aufgaben zugeordnet oder bisher zurückgestellte Arbeiten realisiert. In einzelnen Bereichen gab es große **Schwankungen im Kundenbedarf**, so dass teilweise der Beschäftigungsumfang innerhalb kürzester Zeit von der Kurzarbeit bis zu Überstundenregelungen innerhalb eines Unternehmens ausgereizt wurde.

Die **Einschränkung der persönlichen Kontakte** wurde vor allem im Endkundenbereich negativ bewertet. Diese Aussage trifft insbesondere auf Kundengruppen zu, denen der Zugang zu online angebotenen Leistungen aufgrund fehlenden Internetzugangs nicht möglich war. Die Interaktion mit Lieferanten und bekannten Kunden hingegen war auch unter Pandemiebedingungen in ausreichendem Maße gegeben.

Für die Entwicklung und Umsetzung von Maßnahmen zur Umsetzung der Corona-Verordnungen der Länder und der SARS-COV2-Arbeitsschutzverordnung wurde in den meisten Fällen frühzeitig ein **Krisenstab oder Pandemiestab** gebildet. Diesem gehörten neben der Geschäftsleitung weitere Führungskräfte und, wo aufgrund der Unternehmensgröße gegeben, auch Arbeitsschutzfachleute bzw. Betriebsärzte sowie Verantwortliche aus relevanten Unternehmensbereichen an. Als zielführend erwiesen sich das abgestimmte Verhältnis von **globalen Entscheidungen und bereichsspezifischen Regelungen** sowie die **konsequente Terminsetzung **für die Umsetzung der Maßnahmen.

Auch wenn vorhanden, waren die Betriebs‑/Personalräte nicht Teil des Krisenstabes, aber entsprechend der Mitbestimmungsregeln wurden die beschlossenen Maßnahmen mit der Arbeitnehmervertretung abgestimmt. Teilweise konnte bereits auf bestehende **Betriebsvereinbarungen** Bezug genommen werden, in Einzelfällen entstanden neue Betriebsvereinbarungen.

Der **Austausch mit anderen Unternehmen** bei der Lösungsfindung und der Zugriff auf deren Erfahrungen bzw. auch der unternehmensinterne Austausch von Erfahrungen spielte eine große Rolle bei der erfolgreichen Maßnahmenumsetzung. Die Festlegung von Maßnahmen entsprechend der spezifischen Standort- bzw. Bereichsbedingungen hat sich bewährt. Allerdings waren gerade im Bereich der öffentlichen Verwaltung umfassende Abstimmungsnotwendigkeiten über verschiedene Hierarchieebenen hinweg zu beachten.

Für die Kommunikation der Festlegungen und Maßnahmen wurden u. a. die durch den Krisenstab oder in dessen Auftrag erarbeiteten Organisationsanweisungen genutzt. In einigen Fällen entstanden **Krisenpläne**, die ein Gesamtkonzept zum Gegenstand hatten und sich durch langfristige Adaptionsfähigkeit an sich verändernde äußere Bedingungen auszeichnen. Diese werden von den Unternehmen als zukunftsfähiges Instrument für die Bewältigung plötzlicher Ereignisse wie einer Pandemie angesehen.

Die fehlende Einbeziehung der Beschäftigten in die Maßnahmenentwicklung wurde zwar kritisiert, aber angesichts der umfassenden Kommunikation der festgelegten Maßnahmen und der Möglichkeit zum Feedback und ggf. darauffolgende Anpassung der Maßnahmen akzeptiert.

In einzelnen Fällen wurde von **Eigeninitiativen der Beschäftigten** berichtet, die praktikable Lösungen für die Bewältigung erarbeiteten. Solche Initiativen sind erfolgversprechend, wenn die Beschäftigte über die entsprechenden Kompetenzen und Motivation verfügen und sie auf eine bestärkende Resonanz im Unternehmen stoßen.

Entscheidend für den Erfolg der Maßnahmen und Festlegungen war die offene und umfassende Kommunikation innerhalb der Unternehmen und Institutionen. Es wurden, auch parallel, sehr verschiedene **Kommunikationsformen** genutzt. Das Spektrum reicht von persönlicher bis zu IT-unterstützter asynchroner Kommunikation. Die Hauptakteure waren die Geschäftsleitungen und die unmittelbaren Führungskräfte, die nicht nur die Maßnahmen, sondern auch die Hintergründe der Festlegungen kommunizierten. Insbesondere den unmittelbaren Führungskräften kam hierbei eine große Bedeutung zu, da sie auch auf individuelle Probleme reagieren konnten bzw. die Durchsetzung der Maßnahmen in ihrer Hand lag. Mit der Erläuterung von Gründen und der Alternativenauswahl konnten ein höheres Verständnis und positive Einstellung gegenüber dem Unternehmen bei den Beschäftigten erzielt werden.

Unternehmen mit kontinuierlich arbeitenden Krisenstäben überprüften und hinterfragten über den zurückliegenden Pandemiezeitraum die umgesetzten Maßnahmen. Aufgrund des Wellencharakters der Pandemie wurden je nach Verordnungslage die Maßnahmen an die konkrete Situation angepasst. Dem ganzen Vorgang liegt ein **Lernprozess** zugrunde, in dem ausgehend von zunächst intuitivem Vorgehen immer mehr begründete, erfolgsversprechende Maßnahmen fokussiert und die Erfahrungen der vorangegangenen Monate aufgriffen wurden.

Hindernisse für die Umsetzung von effektiven Maßnahmen wurden überwiegend im technischen Bereich benannt. Die anfangs **fehlende technische Ausstattung** mit Hardware und Softwarelizenzen konnte durch Nutzung von privaten Endgeräten abgefedert werden. Das stellte jedoch nicht für alle Bereiche eine gangbare Lösung dar, da dadurch **Datenschutzregelung** ausgehebelt und die Verletzlichkeit der IT-Infrastruktur erhöht wurde. Defizite bestanden auch hinsichtlich der **Befähigung aller Beschäftigten** zum Umgang insbesondere mit neuer Kommunikationssoftware, die eher unsystematisch und sehr individuell abgebaut werden mussten.

## Diskussion

Ziel des Projektes COVID19LL ist es, Maßnahmen und Lösungen zu ermitteln, die in verschiedenen Branchen eingeführt und ggf. modifiziert und optimiert wurden, um während der Pandemie arbeitsfähig zu bleiben.

In den Interviews zeigte sich eine große Vielzahl an kleinen Maßnahmen und Lösungen, die in Summe dazu führten, dass die Unternehmen arbeitsfähig, flexibel und anpassungsfähig blieben. Einerseits ermöglichte ein konsequentes und teilweise pragmatisches Vorgehen eine sehr hohe Reaktionsfähigkeit und -geschwindigkeit auf unvorhergesehene Veränderungen. Andererseits ließen sich keine einzelnen Leuchtturmmaßnahmen identifizieren, sondern das Zusammenspiel der Maßnahmen stellt die eigentliche Neuerung in der Arbeitswelt während der Pandemie dar. Zwischen den Wirtschaftssektoren ließen sich in Bezug auf die umgesetzten Maßnahmen nur geringe Unterschiede feststellen, die explizit auf den jeweiligen Sektor zurückzuführen sind. Vielmehr unterschieden sich die eingeführten Maßnahmen hinsichtlich ihrer Eignung für Bereiche mit verschiedenen Formen der Arbeitsausführung. Aus diesem Grund wurden die Szenarien in die drei Formen der Arbeitsausführung (ortsgebunden, teilw. ortsgebunden, ortsungebunden) eingeteilt und ermöglichen somit den einfachen Transfer relevanter Lösungen oder einzelner Maßnahmen in verschiedene Sektoren und Branchen. Die Relevanz der Maßnahmen und Lösungen für eine Zeit nach der Pandemie wurde auf Basis der Einschätzung der Befragten beurteilt, da die Pandemie zum Zeitpunkt der Veröffentlichung noch nicht abgeklungen ist.

Die Datenerhebung erfolgte über einen verhältnismäßig langen Zeitraum, zwischen Oktober 2020 und Mai 2021, und auch über den Verlauf der Pandemie war die Vielzahl an kleineren Veränderungen und Maßnahmen auffällig. Dies lässt darauf schließen, dass sich die Strategien der Unternehmen, die in der Anfangsphase der Pandemie gewählt wurden, bewährt haben. Auf die Frage, was die Unternehmen in einem nächsten Lockdown anders handhaben würden, wurde auf die bisherigen Erfahrungen im Umgang mit der Pandemie und die aktuell eingeführten Maßnahmen und Lösungen verwiesen, auf die auch zukünftig zurückgegriffen werden soll.

Identifizierte in Unternehmen eingeführte Maßnahmen umfassten, in Übereinstimmung mit anderen Studien (z. B. Krcmar und Wintermann 2020; BMWi 2021), insbesondere die Einführung digitaler Technologien, um ortsflexibles Arbeiten zu ermöglichen. Damit konnte auch in der vorliegenden Untersuchung eine Beschleunigung der Digitalisierung im Kontext der Arbeit (insb. Arbeitsmittel und -prozesse) gezeigt werden. Diese Entwicklung zieht die Notwendigkeit der Umsetzung umfassender Datenschutzmaßnahmen als grundlegende Voraussetzung für ortsungebundenes Arbeiten nach sich sowie flächendeckende IT-Ausstattung und die Definition digitaler Prozesse. Zusätzliche Möglichkeiten digitaler Technologien für eine effektive Kommunikation und Informationsflüsse sollten auch zukünftig genutzt werden und um Flexibilität zu ermöglichen, sollten Meetings vor Ort durch Teilnehmende per Videokonferenz erweiterbar sein.

Wie beispielsweise bei Hofmann et al. (2020), Kunze et al. (2020) und forsa (2020) beschrieben, findet sich auch in den vorliegenden Forschungsergebnissen ein starker Fokus auf der Einführung von Home-Office bzw. ortsflexiblem Arbeiten. Für flexible Arbeitskonzepte stellen eine gute Erreichbarkeit und transparente Kommunikation der Anwesenheit sowie erbrachter Leistung wichtige Erfolgsfaktoren dar. Ein flexibler Wechsel zwischen den Arbeitsorten bietet großes Potenzial die Nachteile reiner Home-Office Lösungen zu vermeiden und gleichzeitig die Arbeit und das Privatleben besser in Einklang zu bringen. Bei flexiblen Konzepten und einem großen Home-Office Anteil sollte der möglichen Spaltung von Teams sowie gegebenenfalls einer Unterbrechung kooperativer Arbeitsbeziehungen gegengesteuert werden. Die Mitsprache und ein hohes Maß an Flexibilität bei der Wahl des Arbeitsortes konnte in Einklang mit Kunze et al. (2020) und forsa (2020) auch in dieser Studie als ein vielseitig gewünschtes Zukunftskonzept identifiziert werden.

Der Wandel in der Führungskultur zeigt sich in den vorliegenden Ergebnissen, beispielsweise in der Notwendigkeit der Sensibilisierung von Führungskräften sowie der Belegschaft hinsichtlich eines bewussten Umgangs mit dem hohen Maß an Eigenverantwortung und Selbstorganisation, die mit flexiblen Arbeitskonzepten einhergehen. Ein gutes Vertrauensverhältnis, welches z. B. auch von Diewald (2020) und Bertelsmann (2020) postuliert wurde, ist in Home-Office Szenarien auch über die Pandemie hinaus von besonderer Bedeutung. Der Aufbau von Vertrauen und emotionaler Verbundenheit benötigt bei rein ortsungebundenem Arbeiten mehr Zeit und sollte daher explizit gefördert werden. Die kontinuierliche, transparente und barrierefreie Kommunikation der Ist-Situation stützt die notwendige situationsbedingte Anpassung getroffener Entscheidungen und realisierter Maßnahmen und kann in Krisenzeiten einen wichtigen Beitrag zu verbesserter Akzeptanz in der Belegschaft leisten. Die Einführung von Maßnahmen sollte konsistent und zuverlässig umgesetzt werden.

### Limitationen

Bei der Einordnung der Studienergebnisse sind einige Limitationen des methodischen Vorgehens zu berücksichtigen. Zum einen kann durch die Durchführung einer begrenzten Anzahl an Interviews nur ein selektiver Einblick in das breite Spektrum an Wirtschaftsbereichen und unternehmerischen Rahmenbedingungen sowie ihrer Wechselwirkungen erlangt werden. Dies gilt auch trotz des gewählten Ansatzes, Gesprächspartnerinnen und -partner aus verschiedenen Wirtschaftssektoren und Unternehmen unterschiedlicher Größen zu akquirieren. Zudem führt dieser umfassende Ansatz dazu, dass Einschätzungen für das Zusammenspiel bestimmter Rahmenbedingungen in einer bestimmten Branche zumeist auf der Perspektive einzelner Befragter beruhen. Dementsprechend sind die Befunde aus den geführten Interviews, entsprechend ihrer Aufbereitung in den entwickelten Szenarien, weniger als eine Differenzierung der Wirksamkeit individueller Maßnahmen zwischen verschiedenen Unternehmen anzuwenden, sondern als Aggregation der gesammelten Erfahrungen zu verstehen, welche durch die vielfältigen beruflichen Hintergründe der Teilnehmenden profitiert. Die hohe Übereinstimmung der inhaltlichen Schwerpunkte in den Interviews ist dabei ein Anhaltspunkt, wenn auch keine Evidenz, dafür, dass die identifizierten Veränderungsschwerpunkte eine Generalisierbarkeit für die deutsche Wirtschaft besitzen.

Als weitere Limitation ist die Zeitspanne der Datenerhebung zu sehen. Durch das Führen der Interviews zwischen Oktober 2020 und Mai 2021 befanden sich manche der Unternehmen zum Zeitpunkt der Befragung noch kurz vor dem zweiten Lockdown, während andere Unternehmen erst gegen Ende des Lockdowns befragt wurden. Aus dieser Differenz ergeben sich zwangsläufig Unterschiede in dem Ausmaß der Erfahrungen mit der Bewältigung der Pandemie sowie ggf. auch in der subjektiven Wahrnehmung der Pandemie durch die Befragten. Des Weiteren war zum Zeitpunkt der empirischen Erhebung die COVID-19 Pandemie noch nicht abgeschlossen. Daher war es für die Unternehmen noch nicht möglich, die Relevanz und Praxistauglichkeit der eingeführten Maßnahmen in einer Zeit ohne krisenbedingte Anforderungen zu erproben. Dementsprechend kann die Zukunftstauglichkeit dieser Lösungen nur anhand erfahrungsbasierter Prognosen der Expertinnen und Experten eingeschätzt werden und noch nicht empirisch in der Praxis untersucht werden.

### Konklusion und Ausblick

Wie erwartet hat die COVID-19 Pandemie in allen befragten Unternehmen zu einer Vielzahl an arbeitsgestalterischen Veränderungen und mit diesen verbundenen Lernprozessen auf allen organisatorischen Ebenen geführt. Im Wesentlichen umfasst dies die Digitalisierung von Prozessen, die Einführung orts- und zeitflexibler Arbeitsweisen sowie die Herausforderungen von Führung auf Distanz, insbesondere während einer weltweiten Krisensituation. In Anbetracht der anhaltenden Pandemie können zum aktuellen Zeitpunkt noch keine belastbaren Aussagen dazu getroffen werden, welche der Maßnahmen auch in einer postpandemischen Zeit einen wertvollen Beitrag zur Arbeitsqualität in den Unternehmen und Organisationen bieten können. Um sich dieser zentralen Frage über die individuellen Einschätzungen und Prognosen der Interviewteilnehmenden hinaus anzunähern, werden die Ergebnisse dieser Studie im Rahmen einer anstehenden Echtzeit-Delphi-Studie weiter evaluiert.
